# Varespladib in the Treatment of Snakebite Envenoming: Development History and Preclinical Evidence Supporting Advancement to Clinical Trials in Patients Bitten by Venomous Snakes

**DOI:** 10.3390/toxins14110783

**Published:** 2022-11-11

**Authors:** Matthew R. Lewin, Rebecca W. Carter, Isabel A. Matteo, Stephen P. Samuel, Sunita Rao, Bryan G. Fry, Philip E. Bickler

**Affiliations:** 1 Division of Research, Ophirex, Inc., Corte Madera, CA 94925, USA; 2 Center for Exploration and Travel Health, California Academy of Sciences, San Francisco, CA 94118, USA; 3 Venom Evolution Lab, School of Biological Science, University of Queensland, St. Lucia, QLD 4072, Australia; 4 Department of Anesthesia and Perioperative Care, University of California San Francisco School of Medicine, San Francisco, CA 94143, USA

**Keywords:** varespladib, inhibitor, LY315920, LY333013, antivenom, ASV, preclinical

## Abstract

The availability of effective, reliably accessible, and affordable treatments for snakebite envenoming is a critical and long unmet medical need. Recently, small, synthetic toxin-specific inhibitors with oral bioavailability used in conjunction with antivenom have been identified as having the potential to greatly improve outcomes after snakebite. Varespladib, a small, synthetic molecule that broadly and potently inhibits secreted phospholipase A2 (sPLA2s) venom toxins has renewed interest in this class of inhibitors due to its potential utility in the treatment of snakebite envenoming. The development of varespladib and its oral dosage form, varespladib-methyl, has been accelerated by previous clinical development campaigns to treat non-envenoming conditions related to ulcerative colitis, rheumatoid arthritis, asthma, sepsis, and acute coronary syndrome. To date, twenty-nine clinical studies evaluating the safety, pharmacokinetics (PK), and efficacy of varespladib for non-snakebite envenoming conditions have been completed in more than 4600 human subjects, and the drugs were generally well-tolerated and considered safe for use in humans. Since 2016, more than 30 publications describing the structure, function, and efficacy of varespladib have directly addressed its potential for the treatment of snakebite. This review summarizes preclinical findings and outlines the scientific support, the potential limitations, and the next steps in the development of varespladib’s use as a snakebite treatment, which is now in Phase 2 human clinical trials in the United States and India.

## 1. Introduction

The effective, accessible, and affordable treatment of snakebite envenoming is an urgent, global unmet medical need [1,2,3,4,5]. It is estimated that more than 5 million snakebites and envenomings occur annually worldwide, causing significant morbidity (with approximately 400,000 snakebites causing permanent deformities or amputation) and mortality (with up to an estimated 138,000 snakebites causing death) [1,2,4]. Disturbingly, it is estimated that >75% of deaths [6] occur outside the hospital setting, highlighting the need for early treatment as well as the current limitations in the safe use and accessibility of antivenoms. Commonly, by the time patients receive the appropriate medical care in a hospital setting, envenoming-related toxicities have progressed such that they can no longer be effectively treated with antivenom [4,7]. Additionally, data suggest that snakebite envenoming is disproportionately severe in childhood with worse outcomes, which is likely due to the intrinsic curiosity and inexperience of children, the large relative mass-dependent venom dose, the volume of distribution, other physiological factors, and the inexperience of physicians in treating pediatric snakebite emergency [7,8,9,10,11,12,13,14,15]. Small, synthetic toxin-specific inhibitors with oral bioavailability could address some of the limitations of antivenom—including near immediate use by lay-people in the event of a snakebite—as well as improve outcomes following definitive treatment in the hospital setting.

The hydrolysis of phospholipids by venom sPLA2s as well as non-enzymatic mechanisms arising from allosteric sites explain the majority of directly induced sPLA2 toxicities [16,17,18,19,20,21,22,23,24,25,26]. As such, the abundant, highly toxic venom enzymes are desirable targets for a small molecule approach. Snake venoms are complex and varying mixtures composed of phospholipase A2s (venom sPLA2s), metalloproteases, serine proteases, alpha-toxins, and other less abundant or infrequent toxins [14,19,24,25,26,27,28]. Of these toxins, venom sPLA2 is generally considered to be the most enzymatically active component of snake venoms, and it exhibits a wide spectrum of pharmacological toxicities that include neurotoxicity, myotoxicity, hemotoxicity, cytotoxicity, cardiotoxicity, homeostatic instability, nephrotoxicity, and edema [14,19]. Venom sPLA2s are present in more than 95% of the world’s venomous snake species as weaponry for predation, defense of predation, and host defense from micro-organisms [14]. They have been implicated as the biological basis for some types of antivenom resistant snakebite or inefficient paraspecificity [19,29,30]. Due to potent enzymatic activity and an ability to diffuse from circulation into interstitial spaces, even venoms with a seemingly low sPLA2 content can be extremely toxic [19,27]. PLA2 enzymes represent a large family of regulatory and defensive enzymes implicated in the activation of the normal, innately defensive inflammatory process, including that which produces multiple organ failure in sepsis [31,32,33]. Neurotoxic venom sPLA2s can block neuromuscular transmission in vertebrate skeletal muscle, causing acute neuromuscular weakness and flaccid paralysis. In turn, this can result in respiratory depression and death by respiratory failure or obstructive asphyxiation [34]. The hemolytic, anticoagulant, and consumptive effects of venom sPLA2s lead to anemia, hypoxia, acute kidney injury, and difficult-to-treat dysregulation of the coagulation cascade [35,36,37,38,39,40,41,42,43,44]. Other venom sPLA2s can cause instability in blood pressure or destruction of skeletal muscle followed by complications such as kidney failure, although these complications are not entirely unique to sPLA2 [45,46,47,48,49,50,51]. Venom sPLA2s can also induce severe swelling and acute necrosis of skeletal muscle (myonecrosis), leading to permanent tissue loss or amputation, as well as other toxicities in blood, such as myoglobinuria-inducing, hemolytic, and platelet aggregation initiating/inhibiting activities. The combination of hemolysis and venom-induced consumption of coagulation factors can lead to catastrophic bleeding and homeostatic and hemodynamic instability. Further, venom sPLA2s can act as a catalyst for other venom components, such as metalloproteases [33]. Therefore, venom sPLA2s are an important potential target for early pharmacological intervention and as an antivenom adjunct, as identified by the World Health Organization (WHO) and others [4,52,53].

### Varespladib (LY315920) and Varespladib-methyl (LY333013)

**Figure 1:**
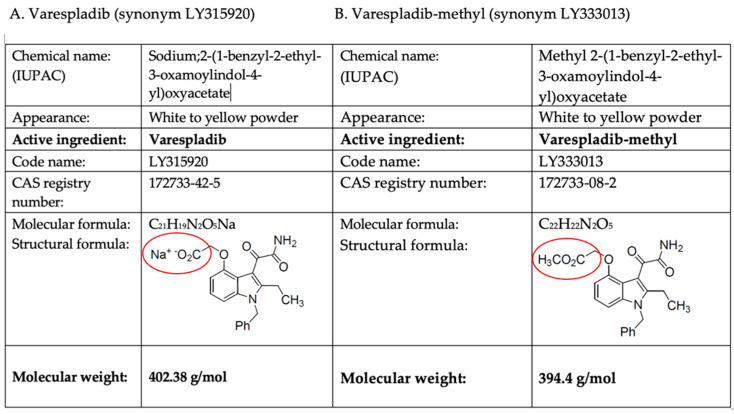
Chemical and structural descriptions of (**A**) varespladib-sodium (API) and (**B**) varespladib-methyl. Circles denote the distinguishing features of the API, a sodium salt and the prodrug, varespladib-methyl which is esterified to facilitate passage through the stomach prior to conversion to the API, varespladib-Na^+^.

The addition of toxin specific inhibitors to the existing standard of care that is underpinned by antivenom could potentially address some significant limitations in the efficacy and the accessibility of current standards of care. Recent observations have shown that the active pharmaceutical ingredient (API) varespladib (synonym LY315920) and its orally bioavailable methyl ester prodrug, varespladib-methyl (synonym LY333013), possess potent inhibitory activity against dozens of venom sPLA2s, with IC_50_ values ranging from pico- to nanomolar concentration in vitro [54]. Varespladib (LY315920) is a substituted indole with the molecular weight of the active pharmaceutical ingredient presenting as a sodium salt at 402.38 g/mol and its prodrug, methylated varespladib, presenting at 394.4 g/mol (Figure 1). Varespladib-methyl (LY333013) studied for snakebite envenoming is presented as a 250 mg tablet with an initial dose of 500 mg, followed by 250 mg twice each day for a total of seven days. In previous human studies, the half-life of the drugs averages eight hours, with Cmax achieved in approximately 90 min, but based on preclinical studies featured in this review, there may be very different pharmacokinetic/pharmacodynamics based on the ultrastructure of the venom sPLA2s.

Varespladib and varespladib-methyl were originally designed as inhibitors of dominant inflammatory sPLA2 isoforms, such as type II sPLA2 (e.g., IIa) and, to a lesser extent, isoforms V and X, and they act as anti-inflammatory agents by disrupting the first step of the arachidonic acid pathway [32,55,56]. To date, twenty-nine clinical studies evaluating the safety, the pharmacokinetics (PK), and the efficacy of non-snakebite envenoming conditions in more than 4600 human subjects have been completed in Phase 1, Phase 2, and Phase 3 studies. These development efforts were led by Shionogi & Company, Limited (Osaka, Japan), Eli Lilly Company (Indianapolis, IN, USA), and most recently, they were abandoned by Anthera Pharmaceuticals, Inc. (Houston, TX, USA). Several studies evaluated efficacy in indications related to ulcerative colitis, rheumatoid arthritis, asthma, sepsis, acute coronary syndrome, and acute chest syndrome associated with sickle cell disease Varespladib or varespladib-methyl were generally well-tolerated and considered safe for use in humans in multiple dosing regimens up to 1000 mg daily for up to 12 weeks in duration. Common adverse events (AEs) reported were comparable to the placebo group, and no allergic reactions have been reported, despite several thousand exposures. Similarly, no clinically meaningful treatment-related effects were noted for the biochemical and hematologic parameters other than the modest transient increases in liver enzymes that occurred in a small percentage of subjects [55]. In VISTA-16, which enrolled more than 4500 patients randomized to receive 16 weeks of varespladib. The patients were high-risk cardiac patients with comorbidities (e.g., diabetes, hyperlipidemia, and prior MI or stroke) who presented with unstable angina or myocardial infarction and received either 500 mg of varespladib or a placebo daily. Seventy-four (1.4%) subjects met the all-cause mortality endpoint. All-cause mortality in the varespladib and placebo treatment groups were 41 (1.7%) and 33 (1.5%) subjects, respectively, with no statistical difference between the treatment groups (95% CI 1.25 (0.79–1.98) [*p* = 0.35]). A total of 69 subjects (1.3%) met the cardiovascular mortality endpoint, with no apparent difference between the treatment groups (95% CI 1.16 (0.73–1.87) [*p* = 0.54]). This study gained wide attention in the field because of the potential cardiac signal not seen in any other studies or the two studies of cardiovascular disease that preceded it [55,57,58,59,60,61]. This review focuses on preclinical findings supporting the use of varespladib as an inhibitor of venom sPLA2s in addition to standard-of-care antivenom treatment, either alone or in combination with other synthetic toxin specific inhibitors and antibody platforms. 

Clinically, these drugs have never been studied in the context of snakebite, and much remains to be learned from human studies of varespladib in patients suffering from snakebite envenoming. 

## 2. Key Laboratory Studies of Varespladib and Varespladib-methyl to Inhibit Venom sPLA2

To date, more than 30 preclinical and nonclinical studies support or clarify the potential uses of varespladib to inhibit venom sPLA2s and minimize their toxic effects in experimentally envenomed animals. These studies include in vitro pharmacology, crystallography, and in vivo and ex vivo studies in mouse, rat, pig, and human blood (Table 1). 

The first study reporting on the ability of varespladib to inhibit a broad-spectrum of venom sPLA2s involved in vitro dose–response curves and pilot in vivo mouse and rat studies. Wang and colleagues found that varespladib and varespladib-methyl displayed inhibition of phospholipase activity at nanomolar and picomolar IC_50_ concentrations against 28 medically important snake venoms from six continents. In vivo proof-of-concept studies used single rescue doses of varespladib (from 4 to 8 mg/kg) that showed a striking survival benefit against lethal doses of *Micrurus fulvius* and *Vipera berus* venom and suppressed venom-induced sPLA2 activity in rats challenged with lethal doses of *M. fulvius* venom [54]. Shortly thereafter, different investigators evaluated the inhibitory potential of varespladib against lethal doses of four highly venomous snakes found in China (*Diengkistrodon acutus, Agkistrodon halys, Naja atra,* and *Bungarus caeruleus*), noting that animals treated with the drug were “vibrant,” while controls had succumbed. Additionally, they reported that varespladib appeared to mitigate the hemorrhagic effects of *D. acutus* and *A. halys* venoms injected intracutaneously. In parallel studies exploring the protective role of varespladib on muscle regeneration, mice were inoculated with *D. acutus* venom or a mixture of venom and 4 mg/kg varespladib (or control) in the gastrocnemius muscle [62]. Local injuries—hemorrhage, myonecrosis, ulceration, and systemic damages, including general dysfunction, visceral failure, and inflammatory responses—were observed at 1, 3, 7, 14, and 21 days. The ability of varespladib (4 mg/kg) to protect against hemorrhage, myonecrosis, and systemic toxicities was evaluated by measuring the subcutaneous ecchymosis, the muscle damage, and the biochemical variation in serum enzymes from envenomed mice. The majority of the muscle myonecrosis and hemorrhage were inhibited by varespladib, and varespladib-treated mice recovered rapidly with lesser atrophy and muscle fibrosis. Varespladib treatment significantly inhibited venom sPLA2, with IC_50_ and ED_50_ values of 0.0016 to 0.063 mg/mL and 0.45 to 22.09 µg/g, respectively. In animal models, the severely hemorrhagic toxicity of *D. acutus* and *A. halys* venom was almost fully inhibited after the administration of varespladib. Moreover, signs of edema in gastrocnemius muscle were remarkably attenuated by the administration of varespladib, with a reduced loss of myonecrosis and desmin. Serum levels of creatine kinase, lactate dehydrogenase isoenzyme 1, aspartate transaminase, and alanine transaminase were downregulated, indicating protection from viscera injury [47].

**Figure 2:**
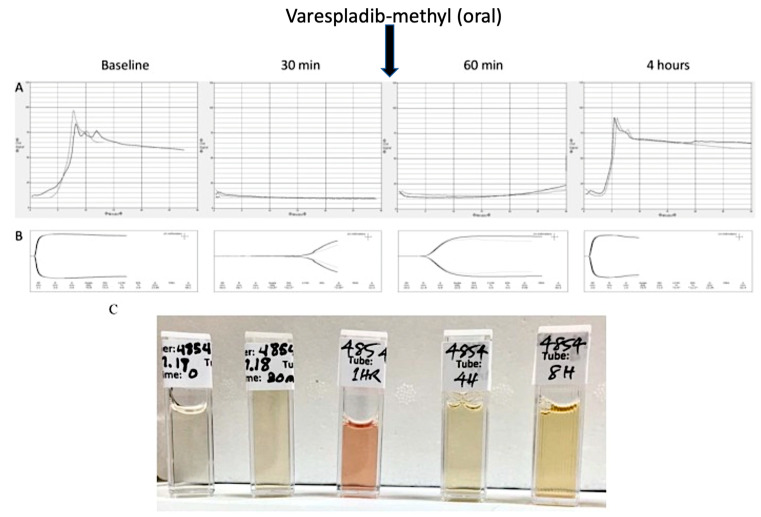
Varespladib-methyl (LY333013) administered following the onset of systemic envenoming signs corrected the anticoagulant effect of *M. fulvius* venom in porcine blood (**A**,**B**). The anticoagulant and hemolytic effects (**C**) often preceded neurological deficits in this study. Others have provided deeper insights into the mechanisms by which *Micrurus* sPLA2 from different regions and in different animals cause more or fewer anticoagulant effects as well as hemolysis. Adapted with permission from Lewin et al. 2018.

Another early key study focused on survival against potentially lethal envenoming. *O. scutellatus canni* was administered subcutaneously, followed by oral administration of varespladib-methyl, CSL taipan specific antivenom, or both after short and long delays. Delayed oral administration of varespladib improved the chances of survival, while antivenom alone did not. However, the combination of drug and antivenom appeared to provide additional survival benefits in this one-week survival study, suggesting some degree of synergy [63]. A subsequent porcine study tested the efficacy of varespladib and varespladib-methyl after lethal envenoming by *M. fulvius*. Inhibitors were administered by either intravenous or oral routes, in the presence or absence of antivenom, and at varying time intervals after venom injection. Each of 14 animals receiving either varespladib (5 mg/kg bolus IV) and/or varespladib-methyl (2.5 mg/kg oral every 6 h or 1 mg/kg oral after 24 h) survived to the 120-h endpoint, despite the presence of severe neurotoxic signs. This study highlighted the capacity of varespladib/varespladib-methyl to protect against, and even reverse, the effects of otherwise lethal envenoming. This was true even against extremely high doses of venom and in cases that were resistant to initial dosing with the antivenom [64]. An appealing element of this study was that there was clear evidence of systemic toxicity at the time of the varespladib administration, as evidenced by the anticoagulant activity seen using thromboelastic techniques (Figure 2). 

The first study to look at the effects of varespladib in human blood exposed to venom was conducted by Bittenbinder and colleagues using innovative robotic techniques to examine spitting cobra-induced anticoagulation and compare the effects of varespladib to antivenom efficiently and precisely [65]. This study evaluated the coagulotoxic effects of 30 African and Asian elapid snake venoms across eight genera. In vitro anticoagulant assays were used to determine the relative inhibition of the thrombin-induced coagulation and the inhibition of the prothrombinase complex formation. This inhibition occurs through competitive binding to a nonenzymatic site on Factor Xa (FXa), thereby preventing FXa from binding to Factor Va (FVa). The authors found that African spitting cobras were the only species whose venom contained potent inhibitors of either clotting factor and Factor Xa inhibition was 12 times greater than thrombin inhibition. At a concentration of 6.25 µg/mL, varespladib potently and completely neutralized the coagulotoxic actions of the African spitting cobra venoms. Similarly, Zdenek et al. showed that while antivenom might not effectively reverse the anticoagulant effects of some elapid venoms, varespladib showed this potential with *Pseudechis colletti*. Further mechanistic insights from purified toxin performed with purified *P. colletti* toxin also show that it has myotoxic properties [66,67]. Coagulopathy and its mitigation in snakebite envenoming is biochemically complex and clinically important; therefore, the inhibitory potential and the limitations of varespladib have gained further attention from groups in the Netherlands, United Kingdom, and India. For example, Alangode et al. found that in blood samples obtained from healthy human volunteers, salts of the fatty acids arachidonic and oleic, and to a lesser extent, linoleic acid, enhanced fibrinogenolysis by Russell’s viper venom (RVV) proteinases [68,69]. In addition to enhancing fibrinogenolysis, sodium arachidonic acid and oleic acid were found to partially inhibit thrombin-induced, factor XIIIa (FXIIIa)-mediated ligation of fibrin chains. Fatty acid anions and anionic detergents induced conformational changes that rendered fibrinogen more susceptible to proteolysis by RVV proteinases, and RVV-PLA_2_ activity (which produces free fatty acids) was required to render the blood incoagulable in clotting experiments with RVV. These observations suggest a mechanism by which the activity of the highly abundant RVV-PLA_2_ promotes the degradation and the depletion of fibrinogen, resulting in the incoagulable blood seen following RVV envenoming. Critically, in clotting experiments with fresh blood, RVV was found to disrupt normal coagulation, leading to small, partial clot formation, whereas RVV pretreated with varespladib induced rapid and complete clot formation (after 5 min). 

Similarly, a collaborative effort between the Kool and Casewell groups produced several remarkable studies. These studies described the in vitro coagulation effects of varespladib in the presence of venom and systematically explored the effects of drug combinations with varespladib, including metal chelators and peptidomimetic hydroxamic acid derivatives, such as the combination of marimastat with varespladib, which showed synergy between the molecules in the in vivo mouse model discussed below. In successive publications, Xie and colleagues reported that varespladib was capable of inhibiting the venom sPLA2 activity of hemotoxic snake venoms and effectively neutralizing the coagulopathic toxicities (including, most profoundly, anticoagulation) [70,71]. Using nanofractionation and high-throughput techniques, the authors also found some evidence that this inhibitory molecule partially abrogated the procoagulant venom effects caused by different toxin families, specifically toxins from the venoms of *B. asper*, *C. rhodostoma*, * D. acutus*, * D. russelii*, * E. carinatus*, * E. ocellatus* and *O. scutellatus*. Recently, the same authors used similar techniques to examine the ability of varespladib to prevent hemolysis [72]. They examined the hemolytic effects of venom using *C. rhodostoma* and *D. acutus*. They also utilized robotic techniques with nanofractionation to look at cobra venoms, such as *Naja pallida*, * N. nigricollis*, and N. *mossambica*, with results extended, and Kazandjian et al. further characterized the reversal of the anticoagulant effects of *N. nigricollis* not reversed by the metalloprotease inhibitor marimastat [73]. These findings further emphasize the potential clinical utility of varespladib in mitigating the toxic effects of certain snakebites.

## 3. Structural and Purified Toxin Studies

X-ray crystallography and computational studies of the sPLA2-like snake venom toxin (MjTX-II) has been used as a model system to evaluate the mechanistic features of varespladib that enable it to nonspecifically inhibit a broad-spectrum of sPLA2s from different species of venomous snakes. Calcium ions (Ca^2+^) are crucial cofactors for catalysis by sPLA2, and the Ca^2+^ binding loop structure of venom sPLA2s is highly conserved. The four key residues in the active site involved in Ca^2+^ binding include: His48, Asp49, Tyr52, and Asp99. In co-crystal structures, varespladib made specific, high-affinity contacts within the structurally conserved hydrophobic groove and Ca^2+^ binding active site of the venom sPLA2 [22,23,74]. The conformationally-restrained indole backbone and ketoamide substituent of varespladib specifically bound a hydrophobic groove conserved across Group II sPLA2 homologs, suggesting how varespladib might inhibit catalytic phospholipase activity regardless of the species-specific sPLA2 quaternary structure. 

**Figure 3:**
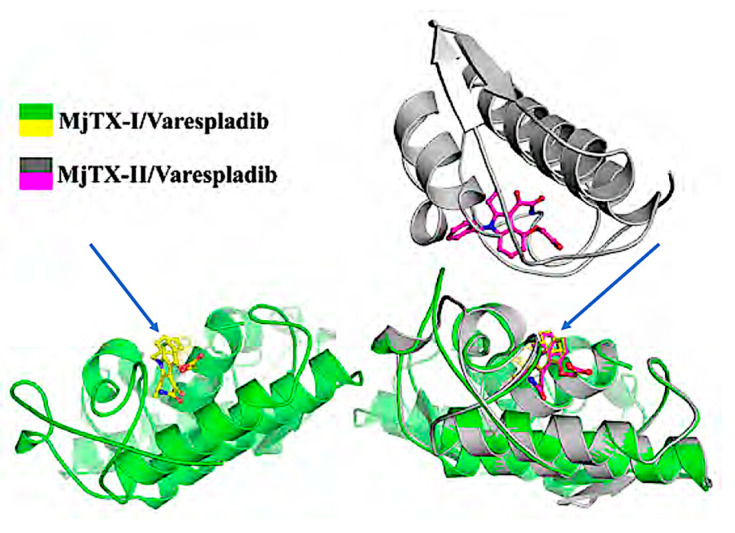
Crystallographic studies examining both the venom sPLA2 and the sPLA2-like toxins from *B. moojeni* demonstrate the binding of varespladib to the hydrophobic channels of these important toxins. MjTx-II in grey (top right with varespladib represented as magenta sticks) and MjTx-I (bottom left, with varespladib represented by yellow sticks) are shown superposed on the bottom right. Arrow point to varespladib bound to the active site [22].

These investigators have additionally examined purified sPLA2 and sPLA2-like toxins to better understand the mechanisms of venom sPLA2 toxicity with and without the presence of varespladib. Two studies by Salvador et al. combined X-ray crystallography and computational techniques with in vitro bioassays to examine varespladib co-crystallized with the enzymatic and non-enzymatic types of *Bothrops moojeni* sPLA2 and the sPLA2-like toxins [22,23] (Figure 3). 

In addition to crystallographic and purely computational studies, such as that of Barbosa [74], there are in vitro and in vivo mechanistic studies evaluating venom sPLA2s of structural group I (pseudexin) and group II (crotoxin B and myotoxin I) and their corresponding whole venoms. Varespladib inhibited the catalytic activity of these three enzymes upon a synthetic substrate in vitro and blocked their cytotoxic effect on cultured murine myotubes [21]. In mice, preincubation of the toxins or venoms with varespladib (at concentrations corresponding to 4 mg/kg), followed by intramuscular injection of the complexes, resulted in the significant inhibition of muscle damage. Finally, immediate, independent injection of varespladib at the site of toxin or venom inoculation also resulted in a large reduction of myonecrosis in the case of pseudexin and myotoxin I and of *P. colletti* and *Bothrops asper* whole venoms. There is much work to be done in this area, and the technical feasibility of co-crystallization of drug and venom is a particularly exciting extension of the work first done on these venoms in the 1990s [18,75,76,77].

## 4. Varespladib and Neurotoxicity in Preclinical Studies

Of special interest regarding sPLA2-driven acute, life-threatening emergencies is neurotoxicity. Neurotoxicity is both clinically important and measurable via observation and quantitative measures, such as those commonly used in the operating room and during recovery. In preclinical studies, purified venom sPLA2s and whole venoms have been used to evaluate the capacity of varespladib or varespladib-methyl to reverse or delay neurotoxic effects. In one demonstration of this principle by Gutierrez et al., mice were injected with the venoms of *Notechis scutatus*, * Crotalus durissus terrificus*, * Bungarus multicinctus*, and *O. scutellatus*, all which had potent presynaptically-acting neurotoxic sPLA2s with differing quaternary structures [78]. A supralethal dose of each venom was injected subcutaneously in the mice, followed by bolus intravenous varespladib or oral varespladib-methyl administration (10 mg/kg), both immediately and at various time intervals after envenoming. The control mice died within 3 h of envenoming. The mice injected with *O. scutellatus* venom and treated with varespladib or varespladib-methyl survived the 24 h observation period, whereas those receiving the *C. d. terrificus* and *B. multicinctus* venoms survived 3 h or 6 h, but not 24 h. In contrast, the mice receiving the *N. scutatus* venom and then verespladib died within 3 h, similar to the control animals injected with venom alone. Varespladib reversed the severe paralytic manifestations in the mice injected with the venoms of *O. scutellatus*, * B. multicinctus*, and *C. d. terrificus*, but it only temporarily staved off the lethality of *N. scutatus*, which also has a potent post-synaptic toxin (3FTx) that can mask effective sPLA2 inhibition in animal models and requires more detailed study to ensure the clinical relevance of animal models where there are combinations of sPLA2 and 3FTx toxins that are undoubtedly important in human and veterinary toxicity. Silva-Carvalho and the Floriano research group at the University of Western São Paulo observed the complexity of sPLA2-3FTx toxidromes in rats envenomed with *Micrurus* where the species had high levels of sPLA2 and 3FTx as their primary neurotoxins. By themselves, varespladib and antivenom had differing partial efficacies to prevent lethality, but in combination, they were surprisingly synergistic in a formal synergy study with varespladib given at very low dose (0.5 mg/kg IP) [79,80]. Similarly, Tan et al. recently observed differing dose requirements in their extreme rescue study of severely weakened and paralyzed mice injected with whole venom from five of Asia’s major krait species (*B. caeruleus*, *B. candidus*, *B. fasciatus*, *B. multicinctus*, and *B. sindanus*). All animals in the study reached severe paralysis stages prior to receiving a single IP dose of varespladib, and an effective reversal of weakness was observed [81]. These studies highlight the challenges of whole animal studies in terms of venom complexity, routes of drug delivery, titration of lethality, and the differential susceptibility of different animals to different toxins, such as rodents, pigs, and humans. These differences raise important questions of interpretation depending on the venom and the model. The differing varespladib dosing requirements (from 10 to 20 mg/kg administered IP) and the durability of the clinical effect may correlate with the 3FTx content in the venom samples, the mice’s susceptibility to this important venom component, and, possibly, the drug composition and the route of delivery. Despite the limitations of using different animal models when complex toxin mixtures are involved, the results from these acute toxicity studies are encouraging and show the therapeutic promise of targeting venom sPLA2 inhibition alone and perhaps with other enzyme inhibitors. 

Early animal studies that focused on acute toxicity (survival) did not address the mechanisms underlying the reversal of weakness. Three additional studies suggest that reversal of weakness occurs through at least two pathways: (1) the restoration of synaptic transmission from the repair of the presynaptic structures and/or removal of the toxin, and/or (2) the restoration of normal neurovascular tone. Oliveira et al., in collaboration with ICP, developed an ex vivo mouse phrenic nerve-diaphragm preparation from which electrodes could measure activity and muscle tone [82]. The ability of veraspladib to abrogate the effect of the potent neurotoxic venom of *O. scutellatus* was assessed using this preparation. Veraspladib (10 µg/mL) inhibited the venom when it was: (a) incubated with venom before addition to the preparation; (b) added to the preparation before the addition of venom; and (c) added to the preparation within 30 min after the addition of the venom, even after the onset of decline in twitch response. This contrasted with previous antivenom results using the same experimental model, in which the effectiveness window was shorter than 10 min. It was proposed that such differences may depend on the higher affinity of varespladib for sPLA2s or the possibility that veraspladib reaches the cytosol of the nerve terminals, inhibiting neurotoxins that have been internalized. Further evidence for a specific mechanism of reversal at the neuromuscular junction comes from two studies using purified venom sPLA2s [83,84]. The venom of the South American rattlesnake *C.d. terrificus* causes an irreversible neuromuscular blockade in isolated preparations due to the action of the presynaptically-acting heterodimeric sPLA2 crotoxin. Studies from UNIS and the State University of Minas Gerais examined the capacity of varespladib to inhibit the neuromuscular effects of crotoxin and its sPLA2 subunit (CB), including a myographic study comparing the neuromuscular effects of crotoxin or CB. Pre-incubation with varespladib abolished the muscle-paralyzing activity of crotoxin and CB and the muscle-damaging activity of crotoxin. The authors concluded that these findings emphasize the clinical potential of varespladib in mitigating the toxic effects of *C.d. terrificus* snakebites [83]. The venom from some populations of this subspecies also contains the toxin crotamine, which directly affects muscle fibers. They applied *C.d. terrificus* venoms with or without the sPLA2 toxin, crotamine, to mouse phrenic nerve-diaphragm preparations previously incubated with two different concentrations of varespladib or antivenom, or with a mixture of these two agents [84]. In another experimental setting, venoms were initially added to the system, followed by the addition of varespladib or antivenom 10, 30, or 60 min after venom. At the highest concentrations tested, varespladib and antivenom inhibited the action of the venom > 80% and >70%, respectively. The inhibition of the neuromuscular blockade decreased with lower concentrations, but when low doses of the two agents were incubated together with the venom, the inhibitory effect improved, underscoring a synergistic phenomenon. When varespladib was added after venom, it was able to halt the progression of the neuromuscular blockade, even when it was added at 60 min. Antivenom exhibited a lower ability to inhibit the toxic effect of the venoms in these conditions. The authors concluded that varespladib is highly effective at abrogating the neuromuscular-blocking activity of crotamine-positive and crotamine-negative *C. d. terrificus* venoms, and it seems to act synergistically with antivenom. Combined, these studies strongly support varespladib action directly at neuromuscular/neurovascular junctions as a potential mechanism for reversing weakness resulting from interruption of the neuromuscular blockade or vascular relaxation [83,84]. 

In the related area of neurovascular toxicity, Vuong et al. examined venom samples from five highly venomous *Pseudonaja* species known to cause cardiovascular collapse in stricken patients, and they had findings suggestive of likely clinical responsiveness to sPLA2 inhibition by varespladib. Collapse due to neurovascular bundle failure at the blood vessel level was examined in ex vivo rat tissue preparations. Pretreatment with varespladib (1 µM) significantly inhibited the vasorelaxation caused by the *P. aspidorhyncha*, *P. nuchalis*, and *P. textilis* venoms. Electrically-induced, sympathetic nerve-mediated contractions of mesenteric arteries were significantly attenuated by only the *P. textilis* and *P. affinis* venoms (30 µg/mL), and these sympatholytic effects were inhibited by varespladib (1 µM). Therefore, it is likely that the venom sPLA2s in *P. aspidorhyncha*, *P. nuchalis*, and *P. textilis* venoms is involved in brown snake venom-induced vasorelaxation and the sympatholytic effects of the *P. affinis*, and *P. textilis* venoms and that envenoming from these species would be responsive to varespladib treatment [45].

## 5. Drug Combinations and Preclinical Animal Models of Complex Non-Neurotoxic Envenoming Syndromes

Not unsimilar to complex neurotoxicity syndromes, hemo-cyto-myotoxic syndromes can occur as the result of exposure to complex tissue destructive venoms with or without neurotoxicity. These types of venom may make it more difficult, but not impossible, to assess single toxin-specific inhibitor approaches. For example, single inhibitor effects in a sPLA2-dominant, mixed-envenoming syndrome were examined by Zinenko et al. in bites caused by Nikolsky’s viper (*V. berus nikolskii)*, which is the only common venomous snake in Ukraine. In this 2020 study, the authors demonstrated that varespladib could prevent lethality in mice envenomed with *V. berus nikolskii*, and they suggested that varespladib would be a feasible alternative to antivenom currently sourced from Russia [85]. In addition, Liu et al. looked at a snake native to Taiwan, *Protobothops mucorsquamatus*. Clinically, *P. mucorsquamatus* shares some similarities to *V. berus nikolskii,* but it has a very different venom composition, with a less than 25% sPLA2 content by weight. Nevertheless, varespladib was shown to reduce the lethal effects of *P. mucrosquamatus* envenoming in a rodent model. In this model, varespladib inhibited the sPLA2 activity in vitro (IC_50_ = 101.3 nM) and blunted the lethality in an in vivo mouse model, prolonging survival following venom injection, but it showed a limited potency against venom-induced local hemorrhage, suggesting that an additional inhibitor, such as a metalloprotease, might be useful [86]. A unique and complex venom from *Lachesis muta rhombeata* was examined by Gutierres. Varespladib abolished the sPLA2 activity of *L. m. rhombeata* venom at low concentrations, but unsurprisingly, it did not inhibit the other major enzymatic groups of this venom (e.g., metalloproteases). Interestingly, though, varespladib (1 mM) alone efficiently prevented the venom (1 mg/mL)-induced procoagulant disorder associated with the extrinsic clotting pathways, and its use together with a commercial antivenom successfully prevented changes in both the intrinsic and extrinsic pathways. Additionally, *L. m. rhombeata* venom (0.5 mg/kg)-induced hemorrhagic activity was slightly reduced by varespladib (1 mM) alone or combined with antivenom (antivenom:venom ratio 1:3 ‘*v/w*’) in rats, although antivenom alone producing no protective action. While varespladib did not inhibit other major enzymatic groups of *L. m. rhombeata* venom, its potent inhibition of sPLA2 activity efficaciously prevented venom-induced coagulation disturbances [87].

To further understand more complex viperid bite syndromes, Albulescu et al. evaluated the efficacy of combining two small molecule inhibitors, including varespladib and the metalloprotease inhibitor, marimastat [88]. This ambitious study looked at both protection and rescue from experimental envenoming in mice that were administered rapidly lethal doses of venom from snakes of subSaharan Africa, India, and Latin America, including *Echis ocellatus*, * Echis carinatus*, * Bothrops asper*, * Bitis arietans*, and *D. russelii*. In a dramatic demonstration of the potential efficacy of combining two small molecule inhibitors with orally bioavailable forms, animals were efficiently and effectively rescued. These results are encouraging and further expand the scope of investigations into treatments intended to reduce death and disability from snakebite envenoming by more than 50% by 2030, as proposed by the WHO [4].

Taken together, these studies demonstrate that varespladib has the potential to:

(1) inhibit the phospholipase activity of venom sPLA2s from a variety of snake species;
(2) block sPLA2 activity in vitro, ex vivo, and in vivo;
(3) provide a therapeutic benefit throughout the time course of envenoming;
(4) protect or reverses neurological damage caused by venom sPLA2s;
(5) restore normal coagulation where anticoagulant sPLA2 are cause of incoagulability or sPLA2-driven consumption coagulopathy
(6) have a synergistic effect with antivenom for many toxicities.

A summary of additional structural, in vitro, ex vivo, and in vivo studies supporting the use of varespladib or varespladib-methyl to neutralize venom sPLA2s is provided in detail in Supplement File S1.

**Table 1:** Studies Evaluating Varespladib (LY315920) and/or Varespladib-methyl (LY333013) in Snakebite Envenoming Models. A comprehensive table of abstracts and links can be found in Supplement File S1.

	Manuscript Title and Link to Abstract	Venoms/Toxins Tested ^a^	Model/Drug & Dosage ^b^	Author/Year
1	Varespladib (LY315920) Appears to Be a Potent, Broad-Spectrum, Inhibitor of Snake Venom Phospholipase A2 and a Possible Pre-Referral Treatment for Envenomation	*Acanthophis antarcticus*, *Agkistrodon blomhoffii brevicaudus*, *A. contortrix*, *A. piscivorus*, *Bitis gabonica*, *Bothrops asper*, *B. jararaca*, *Bungarus caeruleus*, *B. fasciatus*, *Calloselasma rhodostoma*, *Crotalus adamanteus*, *C. atrox*, *C.d. terrificus*, *C. scutulatus scutulatus*, *Dendroaspis polylepis*, *Echis carinatus*, *Laticauda semifasciata*, *Micrurus fulvius*, *Naja atra*, *N. naja kaouthia*, *N. naja*, *Notechis scutatus scutatus*, *Ophiophagus hannah*, *Oxyuranus scutellatus*, *Pseudechis australis*, *Trimersurus elegans*, *Vipera berus*, and *Vipera russelli* (*Daboia*)	In vitro (28 venoms) and in vivo toxicology and pharmacodynamics (mouse/rat) In vitro: 15-point dose–response curves In vivo: LY315920 4–8 mg/kg SC or IV ^†^	Lewin et al., 2016 [54]
2	Exploration of the Inhibitory Potential of Varespladib for Snakebite Envenomation	*D. acutus*, *A. halys*, *B multicinctus*, and *N. atra*	In vitro, in vivo (mouse) LY315920 4 mg/kg SC ^†^	Wang et al., 2018 [62]
3	Inactivation of Venom PLA2 Alleviates Myonecrosis and Facilitates Muscle Regeneration in Envenomed Mice: A Time Course Observation	*D. acutus*	In vitro, in vivo (mouse) LY315920 4 mg/kg SC ^†^	Xiao et al., 2018 [47]
4	Delayed Oral LY333013 Rescues Mice from Highly Neurotoxic, lethal doses of Papuan Taipan (*Oxyuranus scutellatus*)	*O. scutellatus*	In vivo toxicology (mouse) LY333013 10 mg/kg PO	Lewin et al., 2018 [63]
5	Delayed LY333013 (Oral) and LY315920 (Intravenous) Reverse Severe Neurotoxicity and Rescue Juvenile Pigs from Lethal Doses of *Micrurus fulvius* (Eastern Coral Snake) Venom	*M. fulvius*	In vivo toxicology (porcine) LY315920 ^‡^ 5 mg/kg IV bolus, 2.5 mg/kg IV every 6 h and 1 mg/kg LY333013 PO after 24 h	Lewin et al., 2018 [64]
6	Coagulotoxic Cobras: Clinical Implications of Strong Anticoagulant Actions of African Spitting *Naja* Venoms That Are Not Neutralised by Antivenom but Are by LY315920 (Varespladib)	*N. mossambica*, *N. nigricincta*, *N. nigricollis*, and *N. pallida*	In vitro pharmacology (human plasma and fibrinogen) LY315920 6.25 µg/mL ^†^	Bittenbinder et al., 2018 [65]
7	Neutralizing properties of LY315920 toward snake venom group I and II myotoxic phospholipases A2	*P. colletti* and *B. asper*	In vitro and in vivo toxicology, cell culture LY315920 ^‡^ 400 µM	Bryan-Quirós et al., 2019 [21]
8	Structural basis for phospholipase A_2_-like toxin inhibition by the synthetic compound Varespladib (LY315920)	*B. moojeni* ^c^	Crystallography with cocrystallization of LY315920 ^†^ with purified MjTX-II in vitro studies LY315920 ^†^ up to 400 µM	Salvador et al., 2019 [23]
9	Varespladib (LY315920) and Methyl Varespladib (LY333013) Abrogate or Delay Lethality Induced by Presynaptically Acting Neurotoxic Snake Venoms	*B. fasciatus*, *C.d. terrificus*, *N. scutatus*, and *O. scutellatus*	In vivo (mouse) acute toxicity/survival LY315920 ^‡^ and LY333013 10 mg/kg IV, PO	Gutierréz et al., 2020 [78]
10	PLA2 Inhibitor Varespladib as an Alternative to the Antivenom Treatment for Bites from Nikolsky’s Viper *Vipera berus nikolskii*	*V. b. nikolskii*	In vivo (mouse) acute toxicity/survival LY315920^†^ 8 mg/kg SC^†^	Zinenko et al., 2020 [85]
11	Anticoagulant activity of black snake (*Elapidae*: *Pseudechis*) venoms: Mechanisms, potency, and antivenom efficacy	*P. colletti*	In vitro coagulation/hemotoxicity with comparison to antivenom LY315920 2.5 μg/mL ^†^	Zdenek et al., 2020 [67]
12	Varespladib Inhibits the Phospholipase A2 and Coagulopathic Activities of Venom Components from Hemotoxic Snakes	*B. asper*, *C. rhodostoma*, *D. acutus*, *D. russelii*, *E. carinatus*, *E. ocellatus*, and *O. scutellatus*	In vitro coagulation studies (human plasma) LY315920 ^†^ 0.8, 4, 20 µM	Xie et al., 2020 [71]
13	Neutralizing Effects of Small Molecule Inhibitors and Metal Chelators on Coagulopathic *Viperinae* Snake Venom Toxins	*E. carinatus*, *E. ocellatus*, *D. russelii*, and *B. arietans*	In vitro pharmacology human plasma LY315920 ^†^ 4 to 20µM bath	Xie et al., 2020 [70]
14	Sodium oleate, arachidonate, and linoleate enhance fibrinogenolysis by Russell’s viper venom proteinases and inhibit FXIIIa; a role for phospholipase A2 in venom induced consumption coagulopathy	*D. russelii*	In vitro, in vitro, coagulation studies, hemotoxicity (human plasma) LY315920 4 × 10^−3^ mg/mL ^†^	Alangode et al., 2020 [69]
15	Varespladib (LY315920) neutralises phospholipase A2 mediated prothrombinase-inhibition induced by *Bitis* snake venoms	*Bitis cornuta*, *B. xeropaga*, *B. atropos*, and *B. caudalis*	In vitro coagulation studies (human plasma) LY315920 ^‡^ 5.7 nM	Youngman et al., 2020 [89]
16	Varespladib (LY315920) inhibits neuromuscular blockade induced by *Oxyuranus scutellatus* venom in a nerve-muscle preparation	*O. scutellatus*	Ex vivo nerve preparation (mouse) LY315920 ^‡^ 10 µg/mL bath	Oliveira et al., 2020 [82]
17	A therapeutic combination of two small molecule toxin inhibitors provides pancontinental preclinical efficacy against viper snakebite	*E. ocellatus*, *E. carinatus*, *B. asper*, *B. arietans*, and *D. russelii*	In vitro and in vivo (mouse) LY315920 ^†^ 120 µg IV ~6 mg/kg	Albulescu et al., 2020 [88]
18	Anticoagulant *Micrurus* venoms: Targets and neutralization	*M. fulvius*, *M. ibiboboca*, *M. laticollaris*, *M. obscurus*, and *M. tener*	Ex vivo coagulation studies (human plasma) LY315920 ^†^ 10 mg/mL (1% *w*/*v*)	Dashevsky et al., 2021 [90]
19	Snake venom proteome of Protobothrops mucrosquamatus in Taiwan: Delaying venom-induced lethality in a rodent model by inhibition of phospholipase A2 activity with varespladib.	*P. mucrosquamatus*	In vivo toxicology (mouse); LY315920 ^†^ 0.1 mg/kg IP	Liu et al., 2021 [86]
20	Anticoagulant Activity of *Naja nigricollis* Venom Is Mediated by Phospholipase A2 Toxins and Inhibited by Varespladib	*N. nigricollis*	In vitro coagulation (human plasma); LY315920 150 to 450 µM ^†^	Kazandjian et al., 2021 [73]
21	The synthetic varespladib molecule is a multi-functional inhibitor for PLA 2 and PLA 2-like ophidic toxins	*B. moojeni* ^c^	LY315920 ^†^ co-crystallized with purified MJTX-I computational and in vitro studies	Salvador et al., 2021. [22]
22	Action of Varespladib (LY-315920), a Phospholipase A2 Inhibitor, on the Enzymatic, Coagulant and Haemorrhagic Activities of *Lachesis muta rhombeata* (South American Bushmaster) Venom	*L. m. rhombeata*	In vitro and in vivo pharmacology (rat) LY315920 ^†^ 0.001 to 1 µM	Gutierres et al., 2022 [87]
23	Varespladib (LY315920) prevents neuromuscular blockage and myotoxicity induced by crotoxin on mouse neuromuscular preparations	*C. d. terrificus* ^c^	In vitro, ex vivo purified toxin, neurotoxicity, electromyography LY315920 ^†^ 0.25:1, 0.5:1, 1:1 *w*/*w* ratios relative to each purified toxin	Maciel et al., 2021 [83]
24	Role of Phospholipases A2 in Vascular Relaxation and Sympatholytic Effects of Five Australian Brown Snake, *Pseudonaja* spp., Venoms in Rat Isolated Tissues	*P. affinis*, *P. aspidorhyncha*, *P. inframacula*, *P. nuchalis*, and *P. textilis*	Ex vivo (rat) cardiovascular physiology LY315920 ^†^ 1 µM bath	Vuong et al., 2021 [45]
25	In vivo treatment with varespladib, a phospholipase A inhibitor, prevents the peripheral neurotoxicity and systemic disorders induced by *Micrurus corallinus* (coral snake) venom in rats	*M. corallinus*	In vivo toxicology (rat) survival, comparison to antivenom, multiorgan histology LY315920 ^†^ 0.5 mg/mL IP without and with antivenom IV	Silva-Carvalho et al., 2021 [79]
26	Quantum Biochemical Investigation of Lys49-PLA2 from *Bothrops moojeni*	*B. moojeni*	Computational	Barbosa et al., 2021 [74]
27	Varespladib (LY315920) rescued mice from fatal neurotoxicity 1 caused by venoms of five major Asiatic kraits (*Bungarus* spp.) in an experimental envenoming and rescue model	*B. caeruleus*, *B. candidus*, *B. fasciatus*, *B. multicinctus*, and *B. sindanus*	In vivo toxicology (mouse) LY315920^†^ 10 to 20 mg/kg IP	Tan et al., 2022 [81]
28	Effect of the phospholipase A inhibitor Varespladib, and its synergism with crotalic antivenom on the neuromuscular blockade induced by *Crotalus durissus terrificus* venom (with and without crotamine) in mouse neuromuscular preparations	*C. d. terrificus*	Ex vivo neuromuscular preparation (mouse) LY315920 ^‡^ 10 to 30 µg/mL bath	de Souza et al., 2022 [84]
29	Repurposed drugs and their combinations prevent morbidity-inducing dermonecrosis caused by diverse cytotoxic snake venoms	*B. arietans*, *B. asper*, *C. atrox*, *C. rhodostoma*, *E. carinatus*, *E. ocellatus*, *N. nigricollis*, *N. pallida*, *D. russelii*, *B. asper*, *C. atrox*, *C. rhodostoma*, *E. carinatus*, *E. ocellatus*, and *N. haje*	In vitro (cell culture) and in vivo (mouse)	Hall et al., 2022 (in production)
30	Partial efficacy of a Brazilian coralsnake antivenom and varespladib in neutralizing distinct toxic effects induced by sublethal *Micrurus dumerilii carinicauda* envenoming in rats	*M. d. carinicauda*	In vivo (rat) LY315920 ^†^ 0.5 mg/kg IP	Silva-Carvalho et al., 2022 [80]
31	Erythrocyte haemotoxicity profiling of snake venom toxins after nanofractionation	*C. rhodostoma*, *N. mossambica*, *N.nigricollis*, *N. pallida*	In vitro (human RBCs) LY315920 ^†^ 0.4, 8, 20 µM	Xie et al., 2022 [72]

^a^ Venoms from all studies are listed by *Genus* and *species* (Common English Name) below. ^b^ All venoms tested were whole venom, unless otherwise specified with a footnote c. ^c^ Purified toxin. ^†^ Indicates the study used the free acid of varespladib (LY315920∙HCl) that is typically solubilized in DMSO before dilution in the aqueous solution*. ^‡^ Indicates the study used the salt of varespladib-sodium (LY315920-Na^+^) soluble in H_2_O and is API for clinical studies *. Technical Note: The free acid can be converted to the salt by the addition of NaOH, as shown in Supplement File S2. The salt free acid is commercially available from ChemieTek (Indianapolis, Indiana).

## 6. Discussion

Recently the WHO has established a goal to reduce the number of deaths from snakebite by 50% by 2030 [4]. As a major part of meeting this goal, significant focus has been given to evaluating the potential of small molecule therapeutics, such as the sPLA2 inhibitor varespladib and the metalloprotease inhibitors, such as marimastat. No clinical studies of varespladib and varespladib-methyl have yet been completed for the treatment of snakebite envenoming, but foundational laboratory work has been supported by a global collaborative effort to verify, extend, and investigate the potential and the limitations of varespladib. Since 2016, more than 30 separate investigations have been undertaken by groups in China, Costa Rica, Brazil, Denmark, England, the Netherlands, Australia, Ukraine, Thailand, Taiwan, the United States, and India.

In most preclinical studies published to date, two significant technical points have limited their usefulness in interpreting the appropriate clinical dosing pathways, including intraperitoneal venom administration and the requirement of using DMSO to dissolve the free acid form of varespladib. Clinically, varespladib is administered via the IV route as a water-soluble salt [31]. To date, only one research chemical vendor has made the salt form of varespladib commercially available (ChemieTek, Indianapolis, Indiana); however, the free acid, which is widely available, can be converted to the salt in the presence of sodium hydroxide and then diluted for experimental use. While the effects of DMSO can be mitigated by diluting stock solutions, its presence in in vitro studies suggests the need for additional controls. Other areas of significant limitation related to animal and coagulation models are difficulties evaluating mixed toxicities and the propensity for varespladib to precipitate in vitro in the presence of high concentrations of Ca^++^. Different animals have differing susceptibilities to toxins, which is nicely illustrated by the differing susceptibility of different species’ erythrocytes to lyse in the presence of *M. fulvius* venom, for example [91]. This is also particularly notable with neurotoxin studies where the successful blockade of sPLA2s does not necessarily result in survival during studies of acute, lethal toxicity from whole venom, though this limitation is an opportunity for research into mixed pharmacological interventions for snakebite (e.g., an sPLA2 inhibitor with antibodies targeting 3FTx or sPLA2 inhibitor in conjunction with a metalloprotease inhibitors or with, for example, neostigmine, which is an acetylcholinesterase inhibitor.

The strength of support for advancing varespladib to clinical trials includes the independent nature of the investigations and the diversity of in vitro, ex vivo, and in vivo studies ranging from computational biology to mouse, rat, and porcine models. More than sixty venoms have been nominally tested (approximately half were simple enzymatic assays confirming the inhibitory potential of varespladib in vitro), and more than a dozen have confirmed varespladib rescue from acutely lethal envenoming syndromes using whole venom. Several of those trials studied varespladib in combination with antivenom, suggesting both compatibility and the surprisingly consistent finding of synergy [63,64,79]. The BRAVO study is a Phase 2, placebo-controlled study to evaluate the safety, tolerability, and efficacy of varespladib in subjects bitten by venomous snakes. In this study, varespladib-methyl or placebo are administered in addition to the institutional Standard of Care for seven days. The compiled benefit-to-risk profile of varespladib or varespladib-methyl in human subjects has generally been considered acceptable and supports the use of these drugs in the current trial (NCT04996264).

## 7. Future Studies

Some exciting areas of investigation with both basic science and clinical relevance are in neurophysiology (e.g., combinations of sPLA2 inhibition with or without neostigmine or targeted antibodies) and in a more aggressive expansion of the combination studies mentioned above, such as sPLA2 in combination with metalloprotease inhibitors and studies of synergy between small molecules and antivenom. An important and challenging area for future research relates to non-lethal toxicities, as local tissue injury resulting in deformities, contractures, and amputations is far more common than lethality and can be economically devastating. To that end, venoms should not be judged by lethality alone. Long-term laboratory studies of myotoxicity and other tissue damage are critically important, but they must be carefully assessed for proper animal care to prevent suffering. Investigations using cell-based techniques are desirable for laying these foundations and dissecting areas of pharmacological promise using direct toxin inhibitors and their limitations. In addition to preventing mortality from snakebite, improving the long-term outcomes of the survivors of snakebite envenoming is a critical dimension of the 2030 Roadmap goals [4].

## 8. Conclusions

Understanding how different envenoming syndromes are affected by current and emerging pharmacological interventions requires great thought and a better understanding of the natural history of these envenoming syndromes and their long-term functional outcomes. To these ends, basic science and clinical studies advancing in parallel provide a fertile environment to increase understanding of varespladib’s fundamental pharmacology and physiology, therapeutic potential, and limitations. In addition, varespladib may serve as a model for the development of other repurposed drugs to expand the toolbox for the care of snakebite envenoming patients. The collaborative international effort of several laboratories has supported advancement to clinical trials that will hopefully result in new building blocks for further clinical studies to better address this ancient, but ongoing, global health challenge.

## Data Availability

The data presented in this study are available in this article and Supplementary Material.

## Supplementary Materials

The following supporting information can be downloaded at: https://www.mdpi.com/article/10.3390/toxins14110783/s1, File S1 [92] and File S2.
