# Supplementary material for: Varespladib in the Treatment of Snakebite Envenoming: Development History and Preclinical Evidence Supporting Advancement to Clinical Trials in Patients Bitten by Venomous Snakes
Source: Toxins (Basel). 2022 Nov 11;14(11):783. doi: 10.3390/toxins14110783 (PMC9695340; doi:10.3390/toxins14110783)
Supplement: Supplementary file 1 [file toxins-14-00783-s001.zip › Supplement File S2_Conversion of Varespladib-HCl to Varespladib-Na.pdf]

**To prepare Varespladib Sodium *in situ* using Varespladib Free acid**

Basic data/information:

Varespladib-HCl (free Acid), MW: 380.4 (Anhydrous)
1 mg equals 2.63 μmol (Active ingredient)

To prepare a concentration of 76.1mg/ml (200mM) sodium salt solution
Protocols:

1. Weigh 76.1mg (200 μmol) Varespladib-HCl, free acid, powder, into a vial
2. To the vial, add a 1.0 ml 0.2N NaOH (Sodium Hydroxide) aqueous solution (200 μmol NaOH) to form a sodium salt of Varespladib. Swirl to dissolve (Should be dissolved instantaneously).

This a very concentrated Stock solution of Varespladid-Na+, ready to use.
